# Deep Q-network for social robotics using emotional social signals

**DOI:** 10.3389/frobt.2022.880547

**Published:** 2022-09-26

**Authors:** José Pedro R. Belo, Helio Azevedo, Josué J. G. Ramos, Roseli A. F. Romero

**Affiliations:** ^1^ Computer Science Department, Institute of Mathematics and Computer Science, University of São Paulo, São Carlos, Brazil; ^2^ Center for Information Technology Renato Archer, Campinas, Brazil

**Keywords:** deep reinforcement learning, deep Q-learning, social robotics, human robot-interaction, human behavior

## Abstract

Social robotics represents a branch of human-robot interaction dedicated to developing systems to control the robots to operate in unstructured environments with the presence of human beings. Social robots must interact with human beings by understanding social signals and responding appropriately to them. Most social robots are still pre-programmed, not having great ability to learn and respond with actions adequate during an interaction with humans. Recently more elaborate methods use body movements, gaze direction, and body language. However, these methods generally neglect vital signs present during an interaction, such as the human emotional state. In this article, we address the problem of developing a system to turn a robot able to decide, autonomously, what behaviors to emit in the function of the human emotional state. From one side, the use of Reinforcement Learning (RL) represents a way for social robots to learn advanced models of social cognition, following a self-learning paradigm, using characteristics automatically extracted from high-dimensional sensory information. On the other side, Deep Learning (DL) models can help the robots to capture information from the environment, abstracting complex patterns from the visual information. The combination of these two techniques is known as Deep Reinforcement Learning (DRL). The purpose of this work is the development of a DRL system to promote a natural and socially acceptable interaction among humans and robots. For this, we propose an architecture, Social Robotics Deep Q-Network (SocialDQN), for teaching social robots to behave and interact appropriately with humans based on social signals, especially on human emotional states. This constitutes a relevant contribution for the area since the social signals must not only be recognized by the robot but help him to take action appropriated according to the situation presented. Characteristics extracted from people’s faces are considered for extracting the human emotional state aiming to improve the robot perception. The development and validation of the system are carried out with the support of SimDRLSR simulator. Results obtained through several tests demonstrate that the system learned satisfactorily to maximize the rewards, and consequently, the robot behaves in a socially acceptable way.

## 1 Introduction

Initially applied predominantly in industrial environments, robotics is increasingly present in residential and domestic environments. Robots are no longer mere tools to become assistants in everyday tasks in direct partnership with human beings. To allow this evolution, the robotics community must address several challenges, in particular, in the area of Human-Robot Interaction (HRI), especially in the sub-area of Social Robotics (Fong et al., 2003).

HRI is a field of study dedicated to understanding, designing, and evaluating robotic systems to interact and be used by humans (Goodrich and Schultz, 2007). Social robotics directs its interest to interactions between humans and robots, establishing a communication similar to that used between humans. The applications of social robotics range from tasks that involve search and rescue to education to assistive robotics and several other applications that require some social skill.

Considering the definition of SPARC Robotics, 2016 “Cognition is the system-wide process that provides an agent with the ability to understand, given only partial knowledge, how things might possibly be, not just now but at some point in the future, and to use this understanding to influence action”. Since robots and humans communicate, this interaction should occur at the same cognitive level. Predicting the future requires the robot to remember the past, so learning is fundamental for all cognitive systems.

It is desirable that social robots can interact with humans by understanding social signals and responding appropriately to promote a “natural” interaction between humans and robots. This ability depends on both interaction skill levels and cognitive ability levels (SPARC Robotics, 2016). Most social robots are still pre-programmed, lacking the ability to learn and update information (Qureshi et al., 2016). More elaborate methods use body movements, gaze direction, and body language. However, these methods generally neglect necessary interaction signals, such as emotional state and more complex social signals. These signals can provide vital information to assess the success of user interaction.

The ability to interpret emotional signals is essential for social interaction (Kessels et al., 2014). Emotions can help in the interpretation of an individual’s internal states and, consequently, help in the prediction of future actions (Breazeal et al., 2016). Emotions are categorized as purely cognitive, the mental representation of an emotional experience includes both motor and visceral components as well as cognitive components (Dantzer, 1993). Thus, robots must recognize and interpret emotional signals and internally map this information to interact with humans.

The use of *Reinforcement Learning* (RL) represents a way for social robots to learn to behave and interact appropriately with humans, following a self-learning paradigm, seeking to maximize some aspect of the interaction. Through computer vision techniques, such as Deep Neural Networks (DNN) or Deep Learning (DL), robots can capture data from the environment in much more detail, abstracting complex patterns of visual information, including automatically extracted advanced social cognition information. Combining these two techniques is known as Deep Reinforcement Learning (DRL).

In this work, we adopted the use of Ekman’s six basic emotions (Ekman and Friesen, 1971), which are expressed through the human face and are independent of culture. These basic emotions are: *happiness*, *fear*, *disgust*, *surprise*, *anger*, and *sadness*. We address the use of these emotions and other social signals so that robots can behave themselves, with a self-learning paradigm, more socially accepted. In this way, we propose a system based on DRL and *Deep Q-Network* (DQN) (Mnih et al., 2015) so that social robots can identify human interactive behaviors and act appropriately considering social signals, such as the focus of attention and emotions, in addition to captured images of the environment. The robot must learn to identify when the human is willing to interact and which action to select for each case from these signals. This architecture is proposed in this article and is called *Social Robotics Deep Q-Network* (SocialDQN).

Further, we will present the results obtained through training the proposed model to demonstrate its efficiency. We used the *Simulator for Deep-Reinforcement Learning and Social Robotics* (SimDRLSR) developed by Belo and Romero (2021) to assist in the SocialDQN training. This simulator aims to provide an environment for training and validating reinforcement learning systems aimed at HRI and social robotics.

This article is organized as follows. In Section 2, a panorama is presented about works related to the research involving reinforcement learning, use of emotions, social signals, and HRI. In Section 3, some technical and theoretical concepts used in this work are described such as RL, DQN for HRI, and emotions. In Section 4, the proposed architecture, SocialDQN, is presented as well as the emotions considered, mapping the environment, rewards used, actions, among other aspects. The SimDRLSR simulator, used to assist this work, are presented in Section 5. Experiments and results involving SocialDQN and SimDRLSR simulator are shown in Section 6, as well as a brief discussion in Section 7. Finally, in Section 8, conclusion and future work are presented.

## 2 Related works in DRL for social robotics

Modeling the complex social norms established among humans defines a significant research challenge for Social Robotics.

An essential area for social robotics consolidation is cognitive science area that seeks to build models that represent the complex dynamics present in development and human relationships involving characteristics such as perception, memory, mental representation, reasoning and action choice (Abrahamsen and Bechtel, 2012).

Breazeal (2003) reinforced the importance of emotions in social interaction from experiments with a robotic head, named Kismet, capable of expressing emotions through movements in facial features, such as eyelids, eyebrows, lips, and ears. The catalyst for social engagement occurs with the recognition of human movements with the use of cameras. In fact, one of the challenges of social robotics is to merge the interests of the robotic system with information from the environment to define the robotic agent action.

In this direction, research involving social learning gains relevance in contrast to the use of rigid pre-programmed procedures. In the line of social learning, Boucenna et al. (2014) presented how learning by imitation can be used to generate complex behaviors such as social referencing (American Psychological Association, 2022). Moving in the same direction as social learning, we have examples of research that seek to model human cognitive development through the continuous learning of the robotic agent carried out through interactions with humans (Boucenna et al., 2016).

A promising approach to modeling social norms consists of strategies for learning from demonstrations (LfD). In particular, we are interested in the Deep Reinforcement Learning (DRL) technique in this work.

A search in Scopus^1^ reveals that this is an incipient area presenting 55 works in the last 5 years (since 2016). Only 19 results are directly associated with the theme, and 36 results do not directly involve HRI: 19 works are directed to robot navigation, 10 are outside the scope of the search, 6 are associated with publications in *proceedings*, and one is duplicated. To follow, the main works found are presented jointly with a discussion about the advantages and disadvantages of each one.

Qureshi et al. (2016) presented an architecture called *Multimodal Deep Q-Network* (MDQN), which allows a robot to learn human-like interaction skills through a trial and error method. The strategy used involves using MDQN to learn the human protocol with the compliance action as an external reward. The robot *Softbank Pepper* (SoftBank Robotics, 2022b) is used, interacting with people through the actions *wave*, *handshake*, *look* and *wait*. In Qureshi et al. (2018) this architecture was expanded by using the facial expression *smile* and *eye contact* as an additional event associated with *handshake*. The authors modeled a conditional action prediction network (Pnet) and an action-value state network (Qnet). This set turned possible the proposal of an intrinsically motivated deep reinforcement learning framework to learn social interaction skills in the real world of scarce rewards.

The use of artificial intelligence, particularly DRL, can represent a questionable facet in HRI by creating expectations and misunderstanding in the actions performed by the agent (Fiske et al., 2019). The lack of understanding of behaviors and interactions by humans and robots compromises collaboration tasks between both agents (Hayes and Scassellati, 2013; Nikolaidis and Shah, 2013; Hayes and Shah, 2017). Based on these questions, Hayes and Shah (2017) presented a set of algorithms and a monitoring system for enabling robotic agents to answer questions about their actions, intentions, or plans. In addition, a mechanism was presented for modeling robot control policies. The advantage of this proposal is to allow non-experts in the field to obtain insights into the operation of autonomous agents, improving expectations about agents’ behavior.

In the same vein as the MDQN architecture proposed by Qureshi et al. (2016), Clark-Turner and Begum (2018) presented a system that uses DRL, especially the DQN method, capable of learning by demonstrations. For this, a teleoperated Softbank NAO robot (SoftBank Robotics, 2022a) and a set of participants were considered in the experiments. The participants had its gaze, voice, and gestures analyzed by the robot in the context of an Applied Behavior Analysis (ABA) for social greeting intervention. By capturing the image and audio sequences, the network analyzed signals and responses from participants by selecting a set of three actions, *Prompt*, *Reward* and *End*. The *Prompt* action correct the response when the participant exhibits behavior that is not socially acceptable in response to a social greeting. *Reward* is responsible for giving a positive reward in response to positive action by the participant. Finally, the *End* action is performed after a correct answer or after the participant has answered incorrectly several times. The model created showed an accuracy of 68.1% during the simulation and achieved similar results in a real environment.

The real-time emotion recognition increases the applicability of HRI systems. The work proposed in Kansizoglou et al. (2019) explored the temporal quality of human emotion in interactive scenarios using two models of Convolutional Neural Networks (CNN) to extract emotional characteristics through audio and video modalities, jointly to a CNN for a fusion of these models. The DNN output is routed to a Long Short-Term Memory (LSTM) network. Dynamic behavior is achieved through a Reinforcement Learning (RL) agent that monitors the hidden state of the LSTM layer and stops the processing of the video according to its internal confidence. According to the author, this integration is advantageous compared to other models by reducing the time of emotion identification.

Finally, the work proposed in Gao et al. (2019) explored the strategy of gathering a group of people respecting social norms. This work proposed a model called *Staged Social Behavior Learning* (SSBL), for consolidating a previous model of approximation strategies using simulation and applying the knowledge generated in experiments with human participants. The model used a DL scheme associated with a reward function that follows the proxemic theory (Hall et al., 1968).

From the works related to the use of DRL in HRI, we conclude that they have in common the fusion of different types of *Neural Networks* (NN) to process the multimodal information of the environment (gaze, speech, gestures, emotion). This fusion alone is not enough to model complex human social relationships. Thus, the works use the LfD and RL strategies with DRL to mimic human behavior. The presented studies used widely simulators to accelerate the learning process. However, despite promising, the results achieved are still far from the concrete use of the systems produced.

The present work contributes to this research proposing the architecture, SocialDQN, and the simulator, SimDRLSR, to accelerate learning and training considering also human emotions as input information for DRL system.

## 3 Models and techniques for social HRI

In this section, we will present the models, techniques, and theoretical foundations used to develop this work. The work done in Qureshi et al. (2016) and Qureshi et al. (2018) proposed a multimodal reinforcement learning architecture in order to provide “Social Intelligence” for humanoid robots. These works use simple social signals such as a handshake, smile, and human focus of attention.

In the present work, we are proposing to include the analysis of human emotions during the interaction. By interpreting these emotions, robots can infer how to approach and interact with humans. In this way, this work presents a relevant contribution, since that it considers also the human emotions expressed through the face for taking an appropriated action.

Reinforcement learning and deep learning systems require thousands of interactions of the robot and the environment to maximize the exploration of states and possible actions and minimize punishments (or maximize rewards) in a given task. This factor can cause wear on the robot and delay the validation of the system. So, using robots interacting directly with people in the training phase of reinforcement learning systems represents a high computational cost solution. To overcome this drawback, we will work with a robot and people in a simulated environment, through *Simulator for Deep-Reinforcement Learning and Social Robotics* - SimDRLSR proposed by us in Belo and Romero (2021).

RL algorithms aim to maximize a reward signal when performing actions considering a given situation (states) and past interactions. In this context, an agent must identify which actions generate more reward without preliminary information using a trial and error strategy. In more elaborate cases, actions can affect immediate and subsequent rewards for future states (Sutton and Barto, 2018).

According to Sutton and Barto (2018), the understanding of RL depends heavily on the concept of state, which works as an input to the policy and value function. The *Markov Decision Processes* (MDP) defines the formal definition of a state, where a state is any information available to the agent about its environment.

**FIGURE 1:**
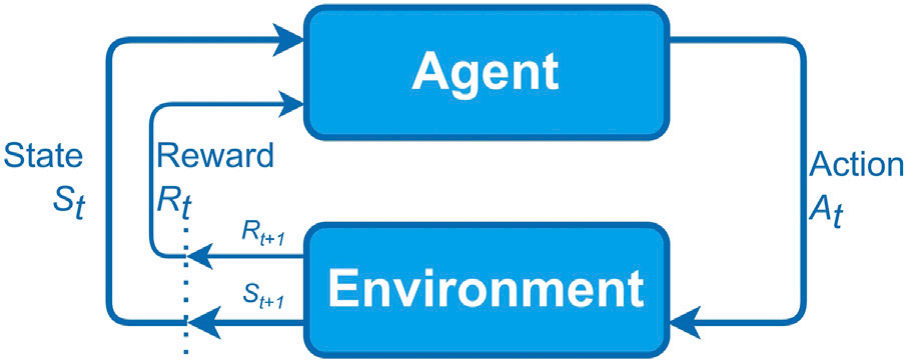
Interaction of an agent with the environment in an MDP (Sutton and Barto, 2018).

In MDP model, an agent interacts with the environment continuously, it selects actions and the environment responds to these actions by presenting new situations to the agent. The environment also generates rewards, values that the agent seeks to maximize over time through the choice of actions. Formally, at each *t* step (*t* = 0, 1, 2, 3, …) the agent receives a representation of the state of the $S_{t}\in S$ environment, and performs an action $A_{t}\in A\left(s\right)$ . When performing an action, the agent receives a reward $R_{t+1}\in R\subset R$ , in addition to the next state *S*_*t*+1_. Figure 1 represents the model of MDP in an agent-environment interaction.

The agent interacts in the environment, accumulating experiences *S*_0_, *A*_0_, *R*_1_, *S*_1_, *A*_1_, *R*_2_, *S*_2_, *A*_2_, *R*_3_ … over time through the MDP. These experiments allow the agent to choose an action *a*_*t*_ from the set of actions A(s) to be executed in the state *s*_*t*_, using a policy *π*(*a*_*t*_|*s*_*t*_) to select the action. The agent uses a policy to map the probability of selecting an action given a state to obtain an estimate of how well this execution performs. The agent then receives a reward *r*_*t*_ and advances to the next state *s*_*t*+1_, given the reward function R(s,a) and the transition probability of state $P\left(s_{t+1}|s_{t},a_{t}\right)$ . This process continues until the agent reaches a terminal state in an episodic problem. At the end of the cycle, is returned the accumulated reward with discount calculated by the discount factor *γ* ∈ (0, 1]. The reward function can be expressed as follows:

$$R_{t}=\overset{\infty }{\underset{k=0}{\sum }}\gamma^{k}r_{t+k+1}$$ (1)

In general, the agent aims to maximize the long-term return expectation for each state. In this way, the action-value function aims to calculate the expected return reward from a state *s*, following a given policy *π*, taking an action *a*. Naturally, at least one policy is always better than or equal to all other policies. For this, Mnih et al. (2015) defined an optimal action-value function Q*(*s*, *a*) as “the maximum expected return achievable by following any strategy, after seeing some sequences and then taking some action *a*”.

For example, the algorithm *Q-learning* (Watkins and Dayan, 1992) updates the optimal policy using policies independent of the policy in question, i.e., the algorithm can use a policy *p* (e.g., random policy) to choose the following action *A*_*t*_ and update *Q* while evaluating the policy *p*′ (greedy policy, for example). This type of algorithm is called *off-policy*. The algorithms *on-policy*, such as SARSA for example, use the same policy to execute and evaluate this policy (Li, 2018).

To enable convergence to an optimal policy, Q-learning has a parameter *ϵ*, which configures an exploration value. In the *ϵ* greed approach, with *ϵ* ∈ (0, 1), an agent selects a greedy action for state *s*, with probability 1 − *ϵ* and selects a random action. This method ensures that the algorithm does not get stuck to a given policy *π*, randomly exploring actions that may or may not maximize future rewards (Sutton and Barto, 2018).

The logic behind algorithms such as Q-Learning and Sarsa is given through the Bellman equation, which according to Mnih et al. (2015), obey the following intuition: “If the optimal value Q*(*s*′, *a*′) of the sequence *s*′ in the next time step has been known for all possible actions *a*′, so the optimal strategy is to select action *a*′ by maximizing the expected value of *r* + γQ*(*s*′, *a*′), from the next equation:

$$Q^{*}\left(s,a\right)=E_{s^{\prime }∼\epsilon }\left[r+\gamma \underset{a^{\prime }}{\max}Q^{*}\left(s^{\prime },a^{\prime }\right)|s,a\right],$$ (2)

However, as the environments become complex, algorithms such as Q-learning may need millions of action-state pairs to map the environment, which is computationally infeasible. Gradient policy optimization algorithms can provide necessary information taking into account sets of actions and states (Sutton and Barto, 2018), being modeled, generally, through *Artificial Neural Networks* (ANN) and DL. Such algorithms learn the policy from a parameter that can select actions without querying a value function. The use of these techniques together with RL, allowed the rise of DRL, mainly by DQN (Mnih et al., 2015).

Mnih et al. (2015) refers to an ANN to model the action-value function Q (Equation 2), as Q-Network. This network is trained by minimizing the Loss function, $L_{i}\left(\theta_{i}\right)=E_{s,a∼\rho \left(.\right)}\left[\left(y_{i}-Q\left(s,a;\theta_{i}\right)\right)\right]$ , that changes at interaction (*i*), where the target for interaction *i* is defined by $y_{i}=E_{s^{\prime }∼\varepsilon }\left[r+\gamma \max_{a^{\prime }}Q^{*}\left(s^{\prime },a^{\prime }\right)|s,a\right]$ , and *ρ*(*s*, *a*) represents the probability distribution over states (*s*) and actions (*a*). From this, the authors present the gradient descent function as follows:

$$\nabla_{\theta_{i}}L_{i}\left(\theta_{i}\right)=E_{s,a∼\rho \left(.\right);s^{\prime }∼\varepsilon }\left[\left(r+\gamma \underset{a^{\prime }}{\max}Q\left(s^{\prime },a^{\prime };\theta_{i-1}\right)-Q\left(s,a,\theta_{i}\right)\right)\nabla_{\theta_{i}}Q\left(s,a;\theta_{i}\right)\right],$$ (3)

where *ɛ* represents samples of environment, and previous iteration parameters *θ*_*i*−1_ are kept fixed when optimizing the loss function. In the context of DQN agents, the model uses this equation to perform gradient descent steps during the training and adjustment phase of the agent’s weights.

Several works have shown the ability of DQN network to learn through high-dimensional visual inputs, working in various areas of robotics, including HRI. For example, in Qureshi et al. (2016), the architecture MDQN is presented for a robot to learn social interaction skills from the experience of interacting with people, which makes use of two DQNs for understanding the environment using high-dimensional visual information. In Qureshi et al. (2018), the authors use a second predictive network in order to calculate the MDQN reward.

In MDQN, the agent interacts for 14 days with the environment for 4 h a day. After each day (episode), the system trains the robot model with the collected experiences. In Belo and Romero (2021), the authors replicated these experiments with the MDQN model using the SimDRLSR simulator with the same training process and interaction conditions.

In the present article, we use several MDQN concepts to propose a new architecture, SocialDQN, that uses human social signals to aid robot learning to promote interactions that are socially acceptable by humans based on their emotional state. For example, this approach includes scenarios where the person does not want to interact with the robot, which must exercise actions relevant to this situation. In addition, we solved the problem of instability in agent training, as observed in Belo and Romero (2021), by generating thousands of episodes and optimizing several parameters.

## 4 SocialDQN: An architecture for social robotics

This section presents the architecture SocialDQN that is the core of this work. This architecture combines social signals from interactions between humans and robots to concepts well established in the literature, such as Q-learning, DNN, and the fusion of these two techniques, the DQN models. The use of images of the environment for training the network represents a way of abstracting complex information from human interaction that may be inherent to cultural and environmental aspects, generating behaviors that are difficult to catalog and classify. However, actual computational methods can easily abstract information from human interaction, such as emotions and facial expressions. This information serves as a tip to the robot on interacting with humans.

**FIGURE 2:**
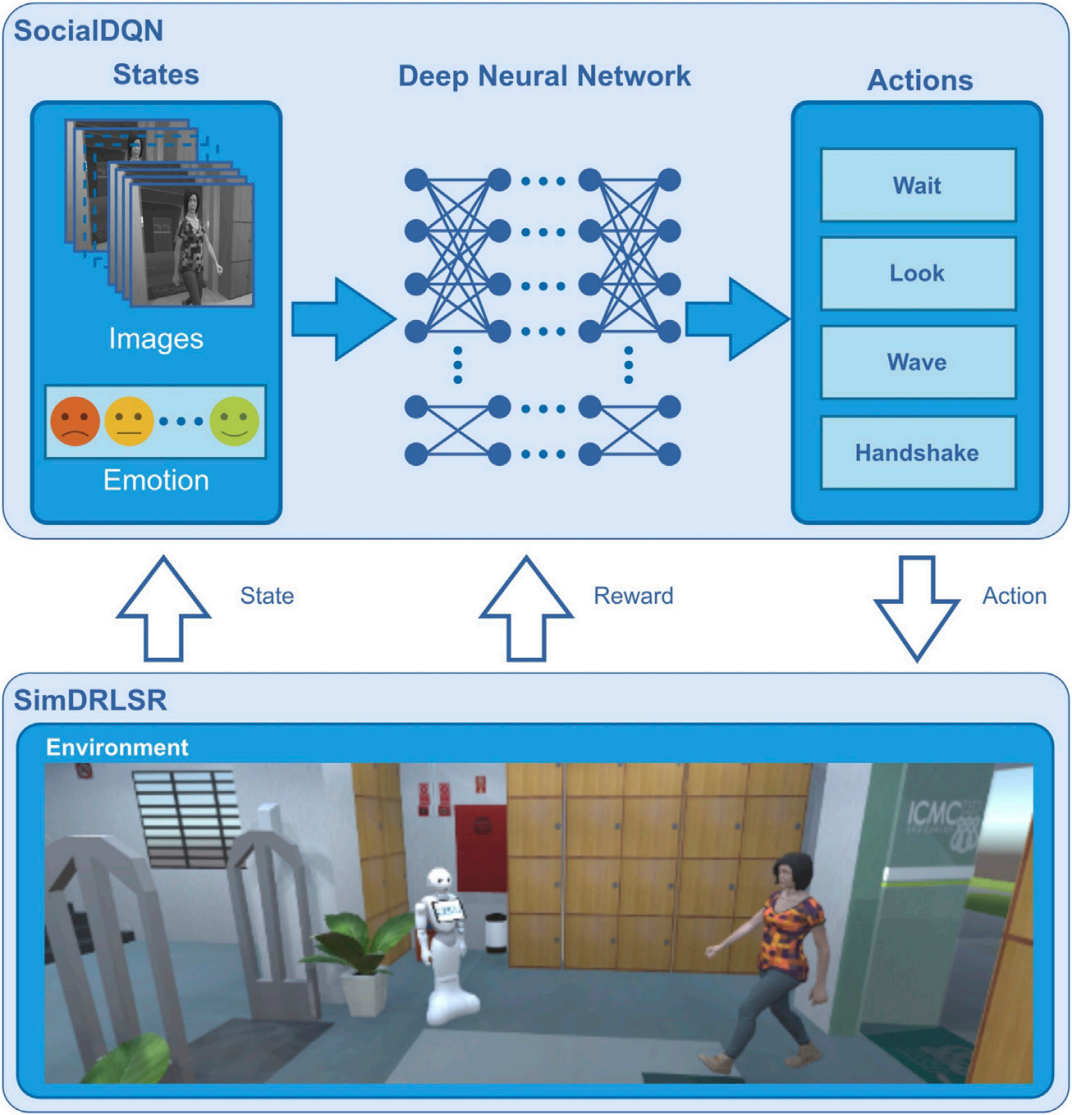
SimDRLSR and SocialDQN.

SocialDQN aggregates experience collected from an environment to train a reference model for teaching the robot to interact in this environment. In Figure 2, it is presented the SimDRLSR (Belo and Romero, 2021), that is the simulator responsible for modeling human, robot, objects, locations, etc. In Section 5 will be presented this simulator in details.

**FIGURE 3:**
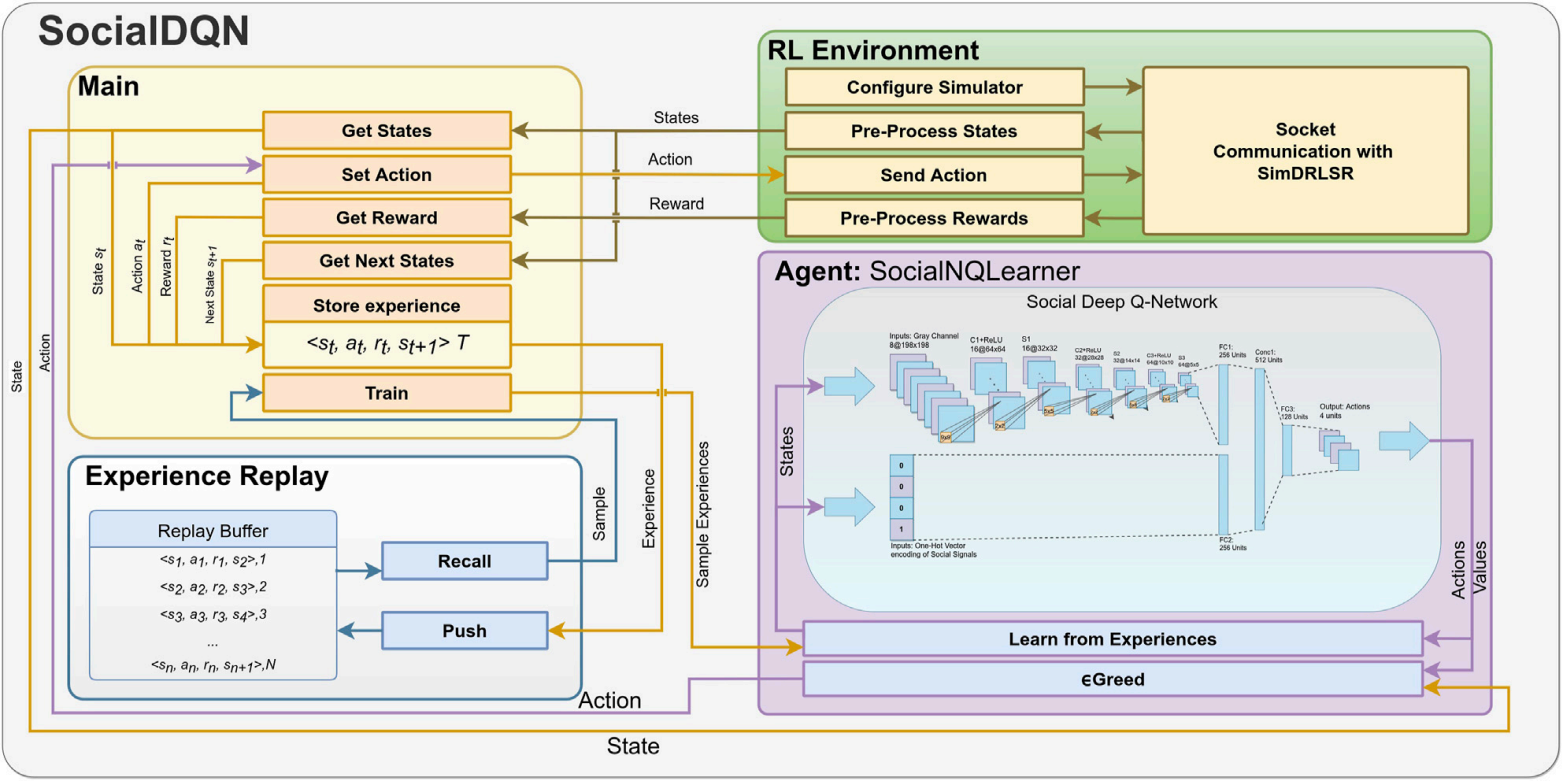
The SocialDQN architecture follows the pattern of a traditional reinforcement learning system. The agent captures the states, selects an action through a policy (*ϵ*-greedy), receives a reward, extracts state information again, and stores the interaction information in a replay buffer. Periodically, the system trains the model. The cycle is repeated until the algorithm reaches a terminal state, and it can restart this cycle for *n* iterations.

The modules composing SocialDQN architecture are shown in Figure 3. They are *RL Environment*, *Agent*, *Experience Replay* and *Main*. In addition, we can observe the states, rewards, actions, and other aspects relevant for training and communication with the robot. In particular, we present the captured states of the environment generated from emotions and grayscale images.

### 4.1 Overview

SocialDQN is a model for the robots to learn, through trial and error, to perform human interaction actions. For this, the agent receives positive or negative rewards depending on its interaction with the environment. This approach allows SocialDQN to assess the quality of its actions in different situations. Unfortunately, robots and computers have sensor and memory limitations to store all the environment information. The abstraction of the environment performed by the agent is known as *state*.

In SocialDQN, a *state* is composed of 8 sequences of grayscale images and a vector with the processed information of human social signals. In this work, the agent uses the emotion detected through the human face to compose this vector. In this way, the agent must use state information to decide what action to take.

For example, given a sequence of images where a human is approaching the robot, and the vector with the social sign informs that the human is happy, the agent shoud decides among to *wave* to get the attention of the human, perform a *handshake*, *look* at him or simply *wait* for him/her to approach. The selected action can generate punishment or reward for the agent.

The agent receives a maximum reward for successfully executing a *handshake* but receives a penalty if the human does not match the gesture. The same happens when the robot waves, but in this case, the agent checks if the human looks back at it, indicating that the robot got the human’s attention.

In addition to sociability issues, the robot must learn the relationship between states and actions to get the human’s attention before establishing a *handshake*. For example, for a person walking in the environment, the robot must wave to him first, and if he approaches, execute a *handshake*.

### 4.2 SocialDQN architecture

This architecture follows the classical structure of a DRL system in which, initially, the agent, the environment, the neural network are created and initialized from parameters and hyper-parameters defined for the execution, training, and validation of the model.

The system starts capturing the state of the environment at time *t*. The *Social Neural Q-Learner* (SocialNQLearner) module retrieves this information, selecting an action from the *ϵ*-greed algorithm. In turn, *RL Environment* is responsible for communicating with the real or simulated robot, being responsible for sending the action generated previously. The system once again captures the state of the environment but in time *t+1*. In Figure 3, the connection arrows between modules represent this continuous movement of information.

Based on the MDQN network, the SocialDQN architecture maps four actions to the robot’s interaction with the environment, *wait*, *look*, *wave*, and *handshake*. In the first action, *wait*, the robot changes its head orientation to look at a random location. In the second, *look*, the robot seeks and maintains its focus of visual attention on a human. The *wave* action involves waving the hand while the robot looks at a human. Finally, the last action, *handshake*, consists in the robot projects its arm forward in order to greet the person. If matched, the robot shakes the person’s hand. We expected that the agent executes each of these actions in the correct context, trying to get the human’s attention until it manages to obtain the highest possible reward from successful execution of the *handshake* action.

The agent stores in *Replay Buffer* the experiences generated from its experience in the environment. Each experiment, associated with execution in time *t*, is represented by the tuple $<s_{t},a_{t},r_{t},s_{t+1}>$ , with the term *s*_*t*_ being the combination of the sequence of eight grayscale images and social signals obtained from the emotion, *a*_*t*_ the action taken for that state, *r*_*t*_ the reward given by the environment, and *s*_*t*+1_ the state at time *t* + 1. The *Experience Replay* stores this experience, overwriting the older ones in case the *Replay Buffer* reaches its maximum size.

This cycle is repeated by the agent collecting information from the environment and performing actions while learning the actions that maximize the reward value. At MDQN network, there was a stage of data collection and training. The data collection stage in MDQN involved long interaction steps before performing the model’s training, with few training episodes. Different from MDQN, SocialDQN trains its model after a few interactions in the environment during thousands of episodes. This aspect ensures a smoother agent learning, as the system constantly trains the model, which allows evaluating and updating the weights present in DQN network and avoiding the agent staying stuck in local minimal.

The SocialDQN is trained by several episodes in which the agent interacts in the environment until it reaches a terminal state. It is considered that the robot reaches this state when it successfully executes the *handshake* action, that is, a human corresponds to this action. The system considers one second type of terminal state. This state occurs when the agent reaches a pre-defined maximum amount of interactions, resulting in a negative reward.

The *Main* module communicates directly to *RL Enviroment*. The latter can have several instances connected to a robot instance (real or simulated). *RL Environment* aims to configure the robotic agent, process states and rewards, and standardize communication with the external system.

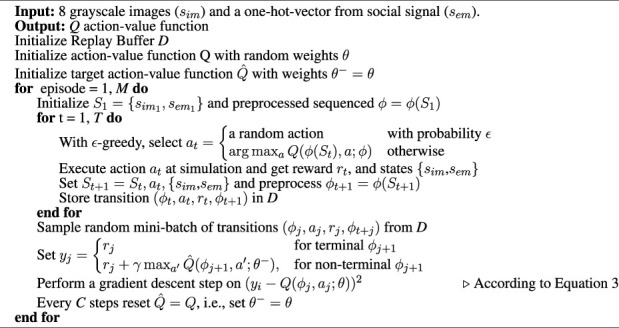

Algorithm 1 SocialDQN pseudo algorithm

In the Algorithm 1 we present the SocialDQN pseudo algorithm, based on the pioneering work of Mnih et al (2015) who proposed the DQNs. The algorithm follows the same execution logic presented above, but it is essential to highlight some points concerning agent training. For standardization purposes with the algorithm presented in Mnih et al (2015), we use *ϕ* as a function to process the input states.

From various experiences in the Replay Buffer, SocialDQN randomly selects a set of a few dozen experiences for training. The training considers the *loss* *Q* concerning the target network $\hat{Q}$ . The *Q* network aims to calculate the quality of values to the execution of an action *a*_*j*_ given the state *ϕ*. The target network processes the future states (*ϕj* + 1), taking into account the action *a*′ that maximizes the network’s return. The SocialDQN trains its model by performing a gradient descent step according to Equation 3.

The training aims to make the network learn to estimate action values from current states concerning future states. The objective is for the agent to learn to estimate its reward when performing an action in a specific state. However, the agent must perform as many interactions as possible, trying to learn the relationship between state and action.

### 4.3 Environment states

A state is a total or partial representation of the environment at a given moment. However, the total representation of an environment can be expensive and requires an enormous quantity of sensors, memory and computational resources to store and process such information. Regardless, the use of images extracted from the environment can bring valuable information to the learning systems, as revealed in several works presented in Section 3.

In the present work, we use sequences of grayscale images as one of the input channels of the DQN network, responsible for mapping the action-state values. The human emotion information captured in time *t* represents the second channel.

In SocialDQN architecture, we only use the grayscale image stream to reduce communication latency, an essential factor in social robotics. Furthermore, grayscale images make training the DQN less complex concerning RGB images. The use of color in deep networks usually occurs when this information contributes to learning. For example, when it is necessary to detect objects, differentiate other robots by color, and identify elements from the environment. In the case of this work, the grayscale images are sufficient to abstract relevant information from the interaction, as shown in many works in the area of HRI (Qureshi et al., 2016, 2018; Clark-Turner and Begum, 2018).

In order to formalize each of these state representations, we will adopt the term *S*_*im*_ for the state represented by the grayscale images and *S*_*em*_ for the mapping of emotional information. The complete set of these two pieces of information at a given time is represented by *s*_*t*_, where *S* is the complete set of all states.

#### 4.3.1 Grayscale image state

We have adopted the same capture parameters used by MDQN architecture (Qureshi et al., 2016, 2018). Thus, we use eight sequences of grayscale images of size 198 × 198, converted from color images of size 320 × 240. Images are captured at a rate of 10 frames per second through the 2D camera on the Pepper robot head.

The MDQN network uses a second image channel to map the depth of the environment. This approach has a high computational cost, both for storing robot interactions in *Experience Replay* and for processing and training these interactions. As a result, the latency time in sending images to DQN network is doubled, generating a delay in the robot’s real-time interaction with humans.

**FIGURE 4:**
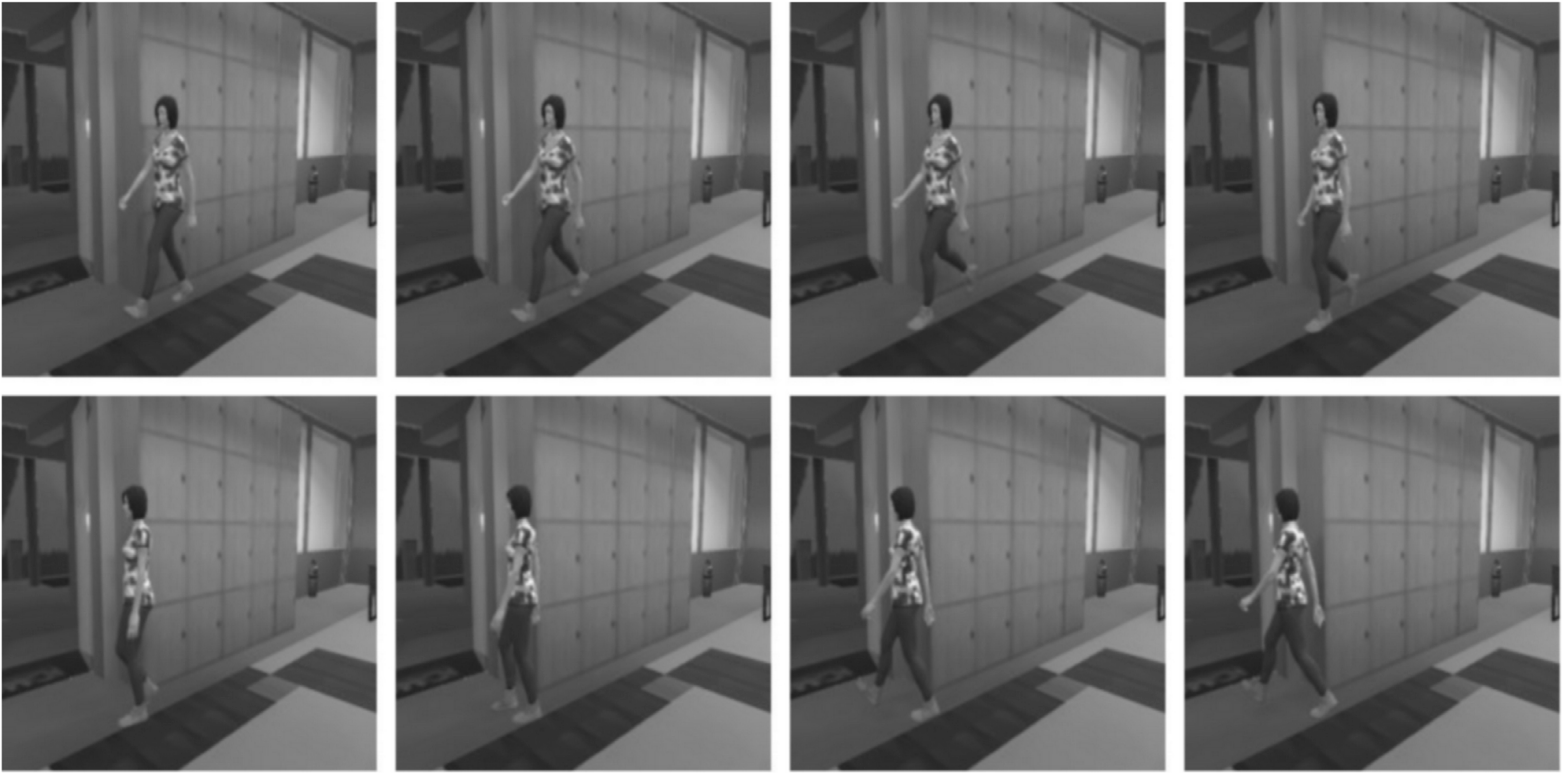
Grayscale images that are part of the state *S*_*im*_. The sequential images help the robot learn according to the direction of human movement. The sequence goes from left to right, top to bottom.

In Figure 4 are shown 8 sequential images that represent a state *S*_*im*_, in a given time *t*. It is possible to visualize a human moving himself in the environment through the images collected by the camera. The DQN network is capable of determining human movement from 8 captured images. Therefore, the robot can inhibit or excite certain actions based on the human’s direction.

In addition to the images, information about the human emotional state is also considered as input to the network. This information is presented to follow.

#### 4.3.2 Social and emotion state

The second component of a *S* state is the processed representation of the human’s emotion detected in the environment. This data concerns the human’s facial expression that is closest to the robot, in the case to have one or more humans in the scene. For this mapping, we used Ekman’s six basic emotions, *happiness*, *fear*, *disgust*, *surprise*, *anger*, and *sadness*.

Human emotion, expressed in the face, is detected by the physical or simulated robot and sent to SocialDQN network through the module *RL Environment* (Figure 3). This approach allows the processing and detection to be carried out in the image capture system, avoiding bottlenecks and delays in the transmission of images to the learning module. As the images of the *S*_*im*_ state have a reduced size (320 × 240 *pixels*), emotion recognition from these images is not feasible, as the facial information represented in the image is usually not sufficient for this detection. A viable solution would be to upload an extra sizable image exclusively for facial recognition. However, some humanoid robots already have emotion recognition system, as well as other social signals, integrated into their operating systems, such as the Softbank Pepper robot (SoftBank Robotics, 2022b), Softbank NAO (SoftBank Robotics, 2022a), and some robotic simulators. We consider that implementing of emotion recognition through the external processing node (robot or simulation) is more advantageous and practical for the proposed system.

During the interaction with the environment, the agent may come across states where there are no humans, or they are not in their field of vision, or even their face is not visible. Thus, we use an additional classification called *No Face* to model a state in which it is not possible to identify the face of a human.

This information is of great importance for learning the DQN network because a *No Face* state may indicate an unlikely human response to any stimulus or robot action in the environment. Otherwise, a visible face may indicate that the human is facing the robot (partially or directly), which increases the chances of the robot being in the human’s field of vision. This information also concerns the presence of a human susceptible to interaction with the robot in the environment. Then, we assume that the *S*_*em*_ state aggregates social signals of emotion and visible human face.

For *Neutral*, *No Face* classes and the six emotions used, the *S*_*em*_ state can currently represent eight possible values. Unlike the *S*_*im*_ state, which aggregates eight sequences of images, SocialDQN uses single information for emotion during that state. There may be several humans with different emotions or even changing emotions during capture in a given scenario. In these cases, SocialDQN network considers the most present emotion in the eight images for a given state.

SocialDQN network maps social signals using *one-hot vector encoding*, which consists of mapping classes into vectors with non-repeating combinations, filled with ‘0’ values and a single ‘1’ value. This mapping can also be represented through an identity matrix. The number 1 in a row position in this matrix indicates the class to which the analyzed emotion belongs.

Despite the use of eight classes for the *S*_*em*_ state, the network used in SocialDQN allows to aggregate or reduce the number of social signals in the system. For example, it is possible to group emotions into *Positive*, *Negative*, *Neutral* and *No Face*, implying in the use of less information about social signals and human emotions. Alternatively, for more complex systems one can add other information, such as social signals from human speech, gestures, number of people in the environment, and other aspects.

### 4.4 Agent and social deep Q-Network

In RL, the *Agent* instance is responsible for receiving information from the environment in the form of states and selecting an action from there. In general, the action leads the agent transit between states. The reward is responsible for helping the agent to choose the action that takes it to a desirable state. In SocialDQN network, the agent is named *SocialNQLearner*.

In *SocialNQLearner*, the DQN network is modeled. This network has two data inputs, one for each of the types of states possible, *S*_*im*_ and *S*_*em*_. DQN is a DL-based (DNN) action-value approximation function, taking as input a state and output a vector *K-dimensional*, in which the *k-nth* element corresponds to the *k-nth* action. This way, the system trains the DQN network to adjust each output value given the expected return.

**FIGURE 5:**
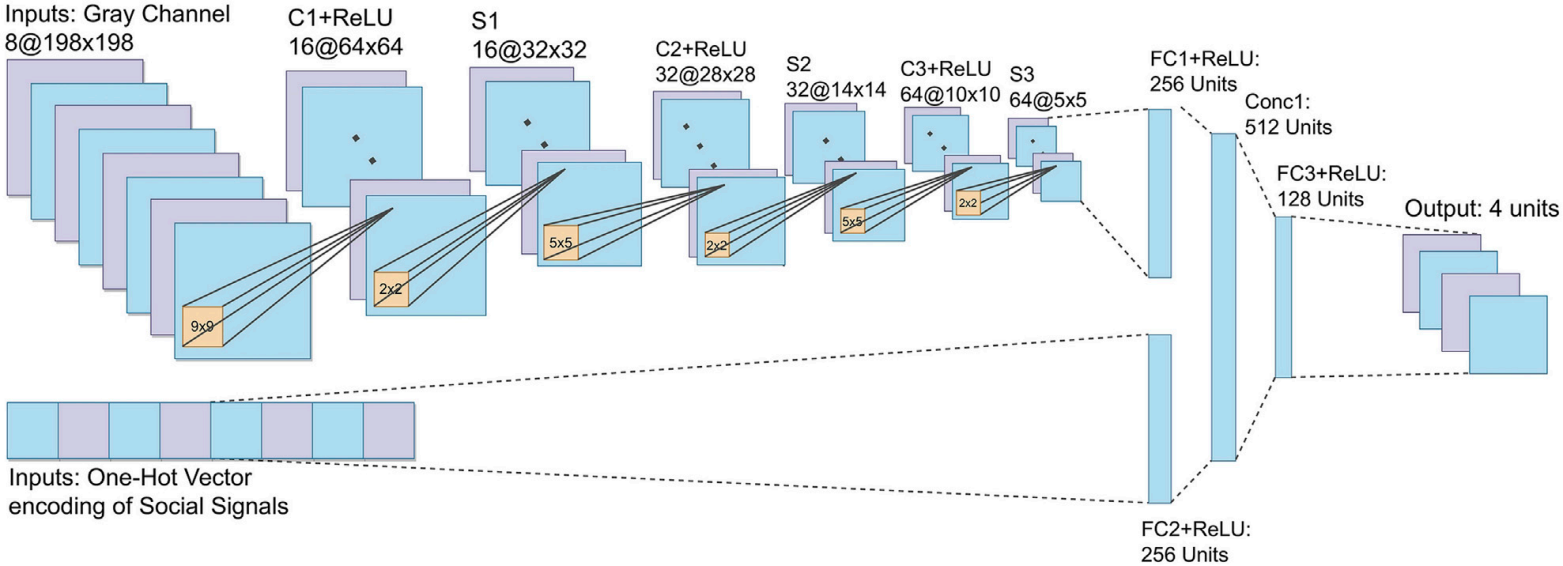
CNN of SocialDQN. The network classifies grayscale images and social signals in the environment interaction phase to generate action values. Each output represents an action, and the action with the highest value is selected. There are two networks identical to the one in the image, a target network and another to be trained in the training phase. Examples of interactions stored in the Replay Buffer are used to evaluate each network, utilizing the reward values obtained in each interaction added to the outputs of the target network to adjust the weights of the main network. The Loss Function performs this adjustment. After *n* iterations, the target network updates its weights with the values of the main network.

The DQN network proposed in this work is presented in Figure 5. The first entry of the network precedes a series of convolution layers and filters responsible for learning the simple and complex visual patterns coming from the *S*_*im*_ states. We adopt the MDQN hyper-parameters for these layers. This input receives eight images of size 198 × 198. They pass to the first convolutional one, where 16 filters of size 9 × 9 are applied. After that, a *Rectifier Linear Unit Function* (C1+ReLU) is used to generate 16 maps of size 64 × 64. In the first sub-sampling layer (S1), the network performs a *max-pooling* 2 × 2 operation from the output of the previous layer. The max-pooling process consists of discretizing a sample to reduce the dimensionality of the data, allowing the network to make assumptions about a sub-region of the data. Layers C2+ReLU, S2, C3+ReLU, and S3 follow the same logic as the previous layers, but now with filters of size 32 and 64, with max-pooling 5 × 5, the output is connected to a linear layer *fully connected* with 256 ReLU neurons (FC1+ReLU).

The second layer receives a *one-hot vector*, responsible for mapping the information of the social signal detected in the environment. The network directs this input to a fully connected layer (FC2+ReLU) with 256 ReLU neurons. This layer and FC1+ReLU are concatenated, forming a layer of size 512, responsible for aggregating the two information flows from the state of the environment. A third and final layer is (FC3+ReLU) fully connected to the output layer with four neurons, each one representing a possible action.

We use the *ϵ*-greedy algorithm for the action selection policy. This approach aims to regulate the execution of random actions versus execution through the DQN network (Sutton and Barto, 2018), as we presented in Section 3. This rate can vary between 1 and 0, decreasing slowly with training. Thus, at the beginning of training, the agent tends to perform random actions to diversify the experiences concerning the action-state. At the end of the training, the objective is to use a lower *ϵ*-greedy value so that the agent applies the knowledge obtained while trying a small number of random actions to improve the reward obtained from the environment.

In the training phase, we follow the training model described by Mnih et al. (2015). In this model, Experience Replay returns a *batch* with a set of random experiences. Randomness in selecting experiences is a feature that the system uses to prevent the agent from learning to correlate sequential experiences.

The Learning phase aims to generalize state-action values, using the reward of the environment to adjust the weight of the network. The local network classifies the states *s* at time *t* (*s*_*t*_) and target network the states *s* at time *t* + 1 (_*t*__+ 1_). As we presented in Section 3, the local network is trained to achieve a given action *a*′ that maximizes the values for the next state in the target network. The idea behind this is to adjust the network weights according to the equation Equation 3 presented earlier concerning a target network.

The target is the estimated value function (local network), used as a target to be reached during the training. This approach aims to make the local network chase the target network values for a few steps, trying to minimize the loss between the two networks. After some iterations, the target network receives the parameters from the main network, the parameters of the target network are frozen, and the process is repeated until the agent ends its training. This strategy is used in DQN to guide and stabilize agent training (Kim et al., 2019).

The *Q* values for the *s*_*t*_ states are updated, according to the expected return, rewards obtained, and a discount factor. After that, the system calculates the value of the *loss* function based on the difference between target network values and the values of the local network. The loss is optimized (Equation 3), and, after *n* training interactions, the system updates the target network to serve as a reference for future training.

### 4.5 Rewards

Rewards are values that the environment generates given the action performed by the agent. The SocialDQN models the reward in different ways. Two of the actions used in our proposal have rewards attached to them.

The *handshake* action generates a positive reward when a human matches the gesture. Otherwise, the environment generates a negative reward. In *wave* action, the goal is to get some human’s attention. When the robot performs this action, the focus of human’s attention is verified. If the focus is the robot, the system generates a reward value. The negative case can occur because no human has been interested in looking at the agent, has its back turned, is busy, or even is not present in the environment. Currently, the other actions do not have rewards linked to them. In this way, SocialDQN assigns neutral values when the agent executes them.

The values for rewards are not fixed and can assume negative, positive, or even neutral values. In the current implementation, we use the value “0” for the positive reward of the *wave* action. This configuration is necessary to prevent the robot from preferring to maximize the total reward by waving several times to the human in sequence, a behavior that is not socially accepted.

In Table 1 the current rewards supported by SocialDQN are listed with their respective values. In the same way as emotions, the events that generate the rewards (touching the robot’s hand, directing the person’s gaze) are captured by the robot or external processing system, and DQN is not responsible for processing these events.

**TABLE 1:** Default rewards for SocialDQN.

Reward Type	Rewards Value
Successfull Handshake	1
Fail Handshake	−0.2
Successfull Wave	0
Fail Wave	−0.1
Neutral (other actions)	0
Fail Episode	−1

It is worth mentioning that works involving MDQN only apply the reward to model *handshake* as failure and success. In Qureshi et al. (2018), a network models this reward, however, the authors did not provide open access to the code and implementation details of this network, and it is not possible to consult details of the prediction network. Therefore, the additional rewards that we defined in this work represent an additional contribution concerning previous works in reinforcement learning and social robotics.

### 4.6 Additional features

SocialDQN was developed in the Python 3.8 programming language, using PyTorch to model and train the DQN network and several other libraries to assist in developing the proposed architecture. The code is available in the GitHub repository through the link github. com/JPedroRBelo/SocialDQN, under the GNU GPL v3.0 license, which allows the community to access the code, being able to modify, publish and share the code freely and to replicate the results presented here.

SocialDQN and the robotic system communicate through a TCP/IP socket, with standardized messages in text format duly converted in each module into their original formats. This technology supports systems with different architectures and operating systems to operate together with ease.

The proposed architecture also allows the customization and configuration of the parameters and hyper-parameters, both the architecture itself and the robotic system. In the latter case, it is possible to change the initialization parameters of a robotic simulator, such as SimDRLSR, for changing the execution speed, screen resolution, size of captured images, graphic quality, and other configurations. Regard to SocialDQN, it is possible to configure several agents, the number of actions, images, social signals, reward values, number of episodes, size of *replay buffer*, network hyper-parameters, such as number of neurons, size of maps, and *max-pooling*, among other countless parameters providing versatility and facilitates training automation.

As it was mentioned, the use of a robotic simulator is advantageous within the scope of SocialDQN, as it speeds up and makes the training and validation process more flexible, allowing control of the environment and system variables and avoiding the wear of the real robot in initial validation tests. Next, we present the SimDRLSR simulator adopted in this worl.

## 5 Simulator for deep reinforcement learning and social robotics

SimDRLSR is a simulator that aims to provide a HRI system development tool with a self-learning paradigm. This tool provides an environment for social robots to learn and identify interactive human behaviors through images and processed social signals. In Belo and Romero (2021), the simulator was initially proposed and used to train the MDQN architecture. It was shown that the simulator can assist in the testing and development phases of social robots for interactions using vision information.

**FIGURE 6:**
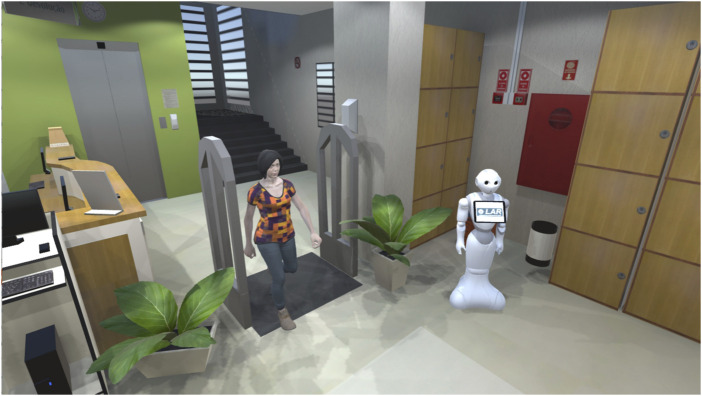
SimDRLSR environment overview. The simulator was developed with Unity Game Engine.

SimDRLSR simulator uses the Unity 3D game engine (Unity Technologies, 2022) for its development. This tool is free and has extensive documentation and a repository where users can share packages (assets) for developing objects on the platform. In Figure 6 is shown the modeled environment, a robot, and a human avatar.

The robot emulated in the simulator is based on the real robot *Softbank Pepper*, respecting the physical limitations of the joints and rotations. It can perform actions, capture images in grayscale depth, and detect events (social signals), initially supporting the needs of MDQN and now SocialDQN.

In Belo and Romero (2021), SimDRLSR maps the four actions presented in Section 4.2: *handshake, wait, look and wave*. An external system controls the simulated robot, sending the actions that the robot must perform. The robot offers grayscale and depth image capture to map the state of the environment. Both images are saved on disk, initially following the limitations of MDQN. However, this approach depends on both systems having access to the same disk limiting independent processing nodes. To avoid this limitation the current versions of the simulator also allow sending, via TCP/IP socket, messages of rewards, actions, and configuration commands.

Further to the images, the robot can detect events and social signals relevant to the system states and rewards. There are three events to detect, *handshake*, human focus, and emotion. The human avatar in the simulation express emotion through the face, which is static throughout the episode.

The simulator models the human behavior through predefined *scripts* and probability tables. As shown in Belo and Romero (2021), *scripts* model a group of commands defined as *Behavior Tasks*. This group establishes which interactions the human will perform in the environment and are independent of the robot. A second group, called *HRI Tasks*, uses probability tables to help model human behavior. This group aims to define actions for the human avatar to interact with the robot. These actions are selected based on several variables such as the robot’s focus of attention and action, interaction distance, engagement, and human emotion.

The SimDRLSR simulator is openly available to the community through the GitHub repository, at the link github. com/JPedroRBelo/simDRLSR, under the GNU GPL v3.0 license.

## 6 Experiments and results

This section presents the results obtained from experiments carried out with a social robot trained with SocialDQN. The SimDRLSR simulates the robot, which aims to support the training and validation of the robot’s learning. Furthermore, we use the results and conclusions of the work carried out in Belo and Romero (2021) to guide the conduction and configuration of parameters in this work.

The presented experiments focus on validating the architecture SocialDQN, aiming to verify the ability of this architecture to provide a framework for training social robots to learn interactive and socially acceptable actions.

The presentation of experiments and results is carried out through the following steps:

1. The system is trained using the simulator that offers an operating environment composed of a human and a robot that interact several times until the training is completed. Details of this step are presented in subsection 6.1 (see Figure 3 *SocialNQLearner*).
2. The analysis of agent learning during training is performed using graphs of the cumulative reward of the process involving 15,000 episodes. In addition, the robot’s behavior is analyzed using graphs that relate emotions and actions. This analysis is presented in subsection 6.2.
3. The trained SocialDQN is exercised during 500 iterations. Subsequently, the SocialDQN is configured to perform random actions without applying the learning performed. A comparison between these two results is performed. Details of this step are presented in subsection 6.3.
4. Finally, the social acceptance of the system is evaluated using a set of human judges who analyzed the behavior of the trained agent. The result of this analysis is presented in subsection 6.4.

### 6.1 Training and learning setup

In Belo and Romero (2021), it was validated the SimDRLSR simulator using the training of the MDQN network as a reference, using the same configuration parameters used in Qureshi et al. (2016). This validation replicates the MDQN network training in 14 episodes, with approximately 2,000 interactions in each episode. However, as demonstrated in the work of Belo and Romero (2021), the training was unstable, that is, it is not possible to reach a state of convergence in the cumulative reward during learning.

Based on this result, we decided to increase the number of training episodes from 14 to 15,000. Additionally, we change the number of interactions in each episode from 2,000 to a maximum of 25 interactions. The episode may end sooner with the robot successfully executing the *handshake* action (terminal state).

SocialDQN trains the model according to Algorithm 1, presented in subsection 4.2. The module SocialNQLearner (Figure 3), learns after each episode when the robot is not interacting in the environment. This configuration ensures that human interaction is not compromised.

We use the *ϵ*-greedy policy for action selection, which starts by selecting random actions with a probability equal to 1 (100%), decreasing over the episodes until reaching the value of 0.05 (5%). As mentioned earlier, the ϵ-greedy approach allows for a balance between the agent exploring the environment and applying the acquired knowledge. This configuration helps the agent to be able to explore the environment beyond the learned situations.

In this work, the emotions were grouped into classes *Positive*, *Negative* and *Neutral*. The first class aggregates the emotions *happiness* (enjoyment) and *surprise*. The second aggregates the negative emotions, which are, *anger*, *disgust*, *fear*, and *sadness*, following the criteria presented according to Tozadore et al. (2018). The third class represents the lack of emotion on the human face. We still use the *No Face* class to indicate when a face is not visible, or there is no human in the robot’s view. Thus, the input vector for the state *S*_*em*_ is of size 4.

The value of the rewards is the same used in Table 1, presented above. The *learning rate* parameter, which defines the degree of adjustment of the network weights, has been adjusted to 25^–5^. Also, we use size 50,000 for *replay buffer* and 64 for *batch size*.

We configured the simulator with a robot and a human per simulation (see Figure 3 RL Environment). At each episode, the simulator resets, repositions of the localization of the human to a random location, and sets the cumulative reward to zero. Then, human emotion is randomly chosen and can assume a positive, negative, or even neutral emotion.

Video 1 (see Supplementary Video S1) presents some interactions of the robot with a human after the training of SocialDQN. In this video the robot executes the wait action in the absence of a human. Soon after, he performs the wave action to gain the human’s attention. Finally, the robot establishes the interaction with a handshake.

### 6.2 Cumulative reward over training

**FIGURE 7:**
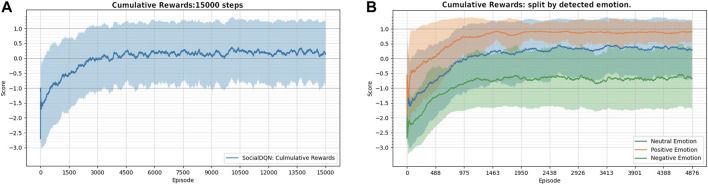
**(A)** the moving average shows the robot’s cumulative reward over the episodes in blue. Light blue spacing represents the standard deviation. **(B)** cumulative rewards split by emotion detected by robot at each epoch.

In Figure 7, in the first graph (A), it is possible to visualize the agent’s learning curve during the training of the network with 15,000 episodes. The graph shows the moving average (dark blue) and standard deviations (light blue). Note that the maximum reward during an episode is ‘1’ because in the ideal case, the episode ends when the agent successfully executes the *handshake*, receiving the maximum reward value. In each episode, the human presents a random emotion. This emotion affects how the robot interacts with the human and can reduce the cumulative reward.

Thus, in the second graph (B), the same cumulative rewards as in the previous graph are presented. However, the episodes are regrouped by the emotions detected (positive, negative, neutral). In SimDRLSR simulator, each type of emotion influences human behavior differently. Note that the robot has a greater chance of success when the human has a positive emotion (orange color) than when it presents a negative emotion (green color). In neutral emotion (blue line), the agent’s behavior is slightly approaching the positive emotion between the two other situations. Note also that the reward curve associated with negative emotion rises with time, indicating that even if the robot is not successful in executing a *handshake*, it learns to avoid performing actions that will reduce the final reward. That is, it learns to maximize the cumulative reward for that situation.

**FIGURE 8:**
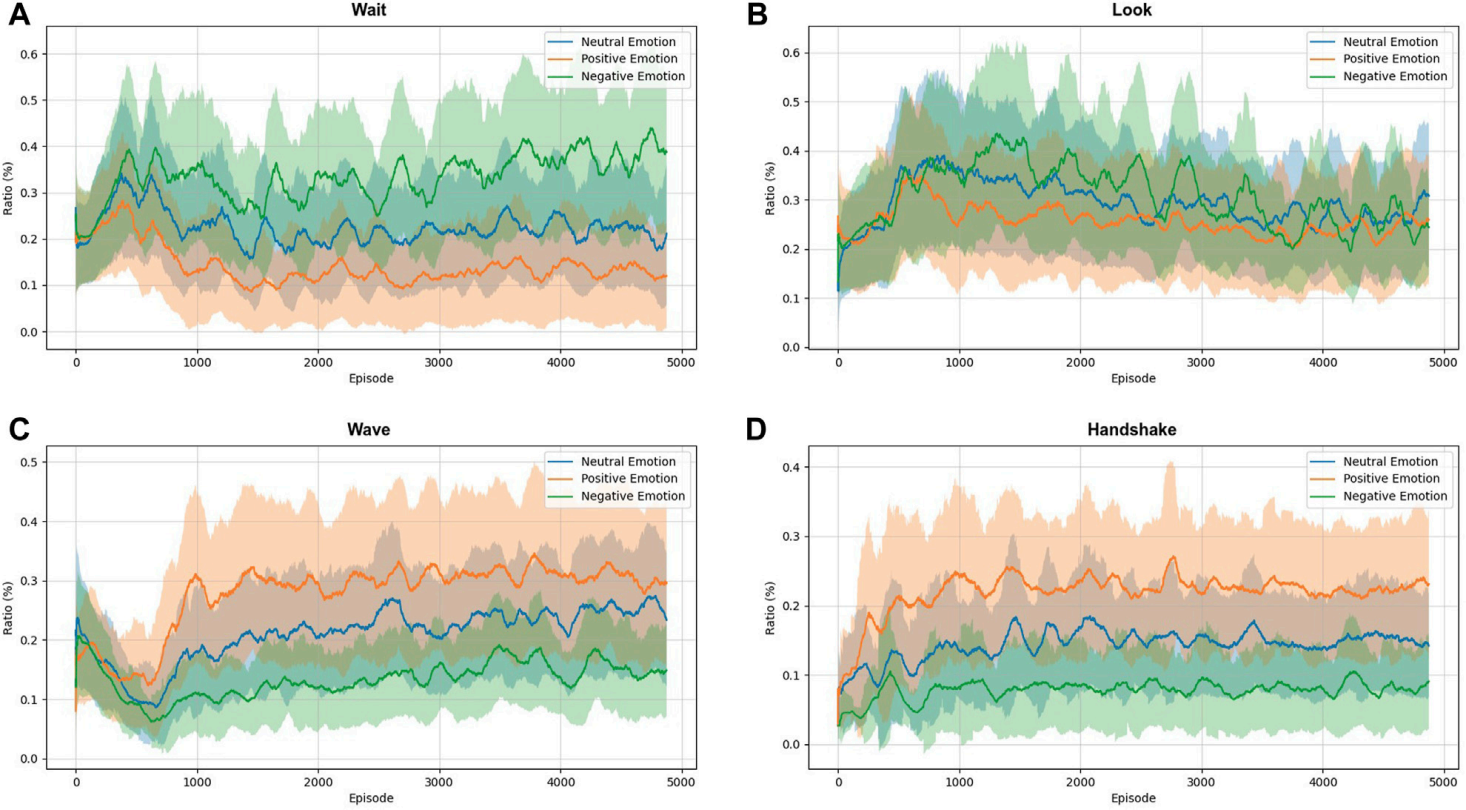
The moving average shows proportion in executing each action concerning human emotion over training. **(A)** Wait; **(B)** Look; **(C)** Wave; and **(D)** Handshake action.

Figure 8 illustrates the proportions in the execution of each action during training, considering the three classes of emotions. The purpose of this figure is to show how the robot learned to behave in the face of different groups of emotions. For example, for negative emotions, the agent tends to perform the actions *wait* (A) and *look* (B). This behavior probably happens because the agent avoids interacting with humans to reduce punishments. For positive emotions, the agent tends to perform one of two actions, *wave* (C) and *handshake* (D), because it learns that in this condition, there is a greater chance of the human responding to its actions. As in the previous analysis, the robot’s behavior is maintained between the other two situations when human is with neutral face.

### 6.3 Validation tests

We performed several tests in the simulated environment after training the network to validate the robot’s learning and verify its behavior. The tests were performed considering two policies for the selection of actions: random and greedy.

In the random policy, the agent disregards learning and the DQN network, selecting randomly one of the four available actions, regardless of the state of the environment. In the greedy policy, the agent selects the action according to the DQN network without using the exploration rate.

The simulator restarts when the robot reaches a terminal state, resetting the position and reallocating another emotion to the human. However, we disregard the negative reward in case an episode ends without the successful execution of a *handshake*. In this experiment, we performed five runs, considering 500 iterations each, for each one of the algorithms.

**FIGURE 9:**
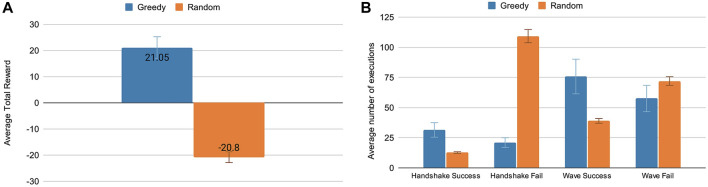
**(A)** Average Total rewards obtained through greedy and random algorithms performed over five runs of 500 interactions each. **(B)** The average number of times handshake and wave actions were performed by the robot successfully or failed over 500 episodes.

In Figure 9, two results are presented, the first is the Average Cumulative Reward (A), and the second presents the average amount of success and failure of the actions *handshake* and *wave* (B). Both results compare the two algorithms used. The first graph shows that the agent has learned to interact in the environment to maximize the reward. In this graph, the greedy algorithm has an average total reward of 21.05 ± 4.22, while the random approach has an average of −20.8 ± 1.53.

In the second graph, we evaluate the actions *handshake* and *wave* because they have rewards attached, which turns possible to verify the success and failure of their executions. In this graph, the *greedy* policy is also more successful in executing the actions than the *random* policy. It holds to note that the frequency of performing these actions, especially the *handshake*, is relatively reduced in the greedy policy compared to the random one. This frequency indicates that the robot tends to be cautious, performing this action only when it considers it opportune.

In the experiments presented, we evaluated the robot’s actions in a quantitative aspect, focusing on the cumulative reward. However, it is interesting to evaluate how humans see the social acceptance of these actions. Thus, to follow we present results from evaluations with human referees (evaluators) to obtain their opinion in relation to the robot behavior with the learning acquired.

### 6.4 Validation of robot actions

This experiment was performed to evaluate whether the robot’s actions are socially acceptable in according to the people’s opinion. The experiment consisted of presenting different scenarios for 13 human referees (people that voluntarily accepted to participate of the experiments) and the action that the robot performed in each one of theses scenarios. Then, the referee must respond to whether or not the action taken by the robot was appropriate. If not, the referee informed the best option for the presented situation.

A scenario is composed of eight images and an emotion captured by the agent. We grouped the eight images to form a 1-s movie. The objective is to reconstruct the robot’s perception during a particular time. In total, 100 scenarios were collected for evaluation by each referee.

The tool used to assist in experiments with referees is available at github. com/JPedroRBelo/validation_tool_socialdqn. We developed this tool in Python3.8 and graphical user interface (GUI) libraries to facilitate the presentation of scenarios to referees. The script also presents the action taken by the robot in that scenario. The referee must answer if this action is socially acceptable or not. Video 2 (see Supplementary Video S2) shows some referee interactions with the tool.

The results presented in Table 2 summarize the scores extracted from the referees’ responses. There were divergent responses among referees in the choice of actions. We considered the most voted response (action) for each scenario. We have also compared the results using two other predictors. The first is a model trained without consider social signals and emotions, only with the sequence of grayscale images, and the second uses a random policy.

**TABLE 2:** Scores from confusion matrix resulted from greedy and random polices. In this table: Acc, Prec, Rec, F1Score, Hands and Avg represent abbreviations of the measures: Accuracy, Precision, Recall, F1-Score, Handshake and Average, respectively.

Class	SocialDQN (%)				Without Social Signals (%)				Action Random Policy (%)			
	Acc	**Prec**	**Rec**	**F1Score**	**Acc**	**Prec**	**Rec**	**F1Score**	**Acc**	**Prec**	**Rec**	**F1Score**
Wait	96.00	92.59	92.59	92.59	77.00	75.00	22.22	34.29	55.00	15.38	14.81	15.09
Look	88.00	100.00	63.64	77.78	62.00	40.00	30.30	34.48	56.00	29.63	24.24	26.67
Wave	83.00	59.26	72.73	65.31	58.00	31.48	77.27	44.74	65.00	21.74	22.73	22.22
Hands	91.00	68.00	94.44	79.07	93.00	92.31	66.67	77.42	72.00	29.17	38.89	33.33
Avg	89.50	79.96	80.85	78.69	72.50	59.70	49.12	47.73	62.00	23.98	25.17	24.33

SocialDQN outperforms the two predictors in all metrics presented, reaching an overall accuracy of 89.50%, the precision of 79.96%, Recall of 80.85%, and F1-Score 78.69%. Note that the accuracy obtained for *handshake* in the model *without social signals* was higher than SocialDQN, but the other metrics for this action show less success concerning the SocialDQN model. As *handhsake* is the action with the highest reward value in negative cases, the network *without social signals* tries to prioritize the accumulation of this reward at all costs, without considering social acceptability. On the other hand, SocialDQN tends to moderate the execution of this action when there is no guarantee of success, executing other actions that are more socially accepted, according to the referees. We can assume that this behavior is a consequence of the social signals used for decision-making.

It holds to note that the *wave* action scored lower than the other actions, with 83.00% accuracy and 59.26% precision. When analyzing the scenarios, we observed that the robot learned to perform this action even when a person is turned away and further away. The referees evaluated that this action is hardly acceptable when there is no person in the scene or a person with the back. In addition, in some scenarios, the referees evaluated that it would be necessary to wait for a human to get closer before performing the *wave* action, especially when the human presented a negative emotion (sad).

The actions *wait* and *look* had the best evaluations with the referees. Overall, it is possible to observe that the referees selected these actions when no one is in the scene, or the human turns their back. The results presented in Table 2 show more robust results than those presented in Belo and Romero (2021). In this last work, which was not using social signals, it is possible to observe that the robot performs the *wave* action too much, even when there was no one in the scene, performing the *wait* and *look* actions minimally. The robustness of these results is given thanks to the refinement of the model using thousands of training episodes. In addition, the use of social signals allowed a more refined understanding of the states.

Finally, from the results obtained, it can be concluded that the use of social signals in the learning process brings benefits to the interaction concerning the social acceptance of the behaviors of the robotic agent.

## 7 Discussion of results

In the previous section, we presented three experiments for testing the performance of SocialDQN. The first experiment focused on the agent training and evolution, the second on the post-training phase, and the third focused on validation through referees.

In the first experiment, it was possible to verify that using social signals, precisely human emotion extracted from the human faces, provides to the agent flexibility in performing its actions. Although training encourages the robot to perform the *handshake* action (to obtain the maximum reward), there are situations in which the human does not want to interact with the robot. Thus, the robot needs to identify these situations and perform actions that minimize punishments. An example of this occurred when the human is sad (negative emotion), which should be associated with a greater likelihood of ignore the robot.

It was also possible to verify that even if a given action provides a positive and immediate reward to the agent, this action can lead him to a state that does not have a maximum global (cumulative) reward. This aspect is one of the advantages of reinforcement learning, that is, the ability to map and query the relationship between actions and states, allowing the agent to anticipate the next states and the estimated final reward.

The second experiment was performed to verify the agent’s ability to maximize the reward comparing two different policies: *greedy* and *random*, and verifying the success of the actions *handhsake* and *wave*. The *greedy* policy represents the knowledge acquired during training and excels over the *random* policy. One can see that the greedy policy successfully performs both actions *wave* and *handshake*.

However, the random policy can not perform the actions very well since some actions depend on the observed scenario, which does not happen when the agent executes them randomly. It is worth to mention that the action *handshake* depends on the execution of other actions, as this requires the human to be in ideal conditions for its successful execution. The *wait* and *look* actions validation requires the additional mapping of rewards associated to them.

In the third experiment, the focus was on validating actions concerning social acceptance with referees. The results obtained were quite satisfactory, especially when compared to the random policy. It is important to evaluate the *wave* and *handshake* actions. They presented lower values of accuracy than other actions. This is due to the fact that they have a greater influence on the environment, being able to abruptly change human behavior and being more susceptible to rejection, unlike *wait* and *look*, which do not directly affect social interactions.

Another critical point is that the system learned to behave according to the behaviors modeled through the SimDRLSR simulator, given through probability tables. These tables are an initial effort to model these behaviors, and it is still necessary to refine these behaviors to reflect natural human behavior. Furthermore, the probabilities favor the humans behave differently, even if the robot performs the same action in the same situation. For example, if the robot waves to a happy human, that human has a 90% chance of moving or looking at the robot, with a 10% chance of ignoring it. Even if the robot performs a correct action in the social aspect, the simulator has a degree of randomness that influences the results of validation tests.

In addition to the above mentioned issues, discussing possible problems in the transition from learning to the real robot is interesting. The simulation is a controlled environment and provides a realistic scenario with people, objects, lighting, and shadows. However, real environments have dynamic elements that are difficult to map in simulation. In addition, image capture, communication, and emotion detection, for example, depend directly on the hardware of the real robot, which can lead to latency in communication between systems and even in the system’s performance process. Knowledge transition is still an open aspect concerning SocialDQN, which we will address in future works. It may be necessary to do a fine-tuning, that is, to train the robot in a real environment from the heavy training in simulation.

In general, in all experiments, the results indicate that the robot learned to interact in the environment appropriately and to maximize rewards, avoiding performing actions that lead to an unfavorable state.

Finally, the use of SimDRLSR allowed the development, training, verification, and validation of the SocialDQN architecture. The training phase was one of the crucial points in which the simulator was essential given the need to stabilize the learning of the model by training thousands of interactions with the environment. This issue is fundamental in RL, as the mapping of *action-values* grows exponentially with the number of actions and possible states, requiring more and more interactions in the environment to refine the agent’s behavior.

## 8 Conclusion and future works

In this work, the architecture *Social Robotics Deep Q-Network* - *SocialDQN* was proposed, which aims to use social signals and image sequences to learn human interactive behaviors. The main contribution of this work was to include the emotion analysis of the human during the interactions with the robot for performing relevant actions.

The learning was possible thanks the *Deep Q-Network* technique that considers high-dimensional sensory data and rewards from the agent’s actions as a reference. The architecture SocialDQN was developed, trained with the deep reinforcement learning technique, and validated with the support of *SimDRLSR* simulator, especially customized by this work, which provides a simulated environment for social robotics.

The results achieved through tests and human referees showed that the system learned as to maximize rewards as to inhibit inadequate actions in the several scenarios, presenting an mean of accuracy of 89.5% for the four classes considered. Other point to be highlighted is the fact that the architecture SocialDQN obtained the best values in all of the metrics considered when compared to other two models: without social signals (only images) and random policy. Therefore, the use of social signals, in particular human emotions, collaborated for that the system behaved in a more natural and socially acceptable way.

It is important to note that SocialDQN and SimDRLSR adhere to the open-source model with the availability of versions via a GitHub repository. This provision encourages the collaboration of other researchers in the evolution of the architecture and also of the simulator.

The main limitation of this work is related to a few actions that the agent can take (*wait*, *look*, *wave*, and *handshake*), implying the fact that the system is applied only to initial interaction between robot and human. By adding new actions to the agent, the system can be trained to execute them and have more complex behavior.

Finally, SocialDQN can support everyday applications, interacting with people in private and public places. This system can be applied in library receptions, events, seminars, nursing homes, schools, hospitals, and other environments, in which the robot must present appropriate behaviors for each situation.

In future works, we will model additional rewards to refine the execution and evaluation of agent actions. We will add new actions, social signals, and memory to the system. Further, *transfer learning* could be used for training a real Pepper robot enabling analysis of the operation in the real world.

## Data Availability

The datasets presented in this study can be found in online repositories. Main architecture: https://github.com/JPedroRBelo/SocialDQN; Validation tool with database: https://github.com/JPedroRBelo/validation_tool_socialdqn; Simulated environment used to validate the main architecture: https://github.com/JPedroRBelo/simDRLSR.

## Footnotes

1 Search string = ((“human robot interaction”) OR (“human-robot interaction”) OR (“social robotic”) OR (“social robotics”)) AND (“deep reinforcement learning”).
